# The sticky truth: how spider predation success depends on their prey's body surface

**DOI:** 10.1242/jeb.249347

**Published:** 2025-05-08

**Authors:** Lucas Baumgart, Sascha Schlüter, Marieke Moog, Annika Schönfeld, Adrian Heß, Florian Menzel, Anna-Christin Joel

**Affiliations:** ^1^ RWTH Aachen University, Institute of Zoology, 52074 Aachen, Germany; ^2^ Johannes Gutenberg-University, Institute of Organismic and Molecular Evolution, 55128 Mainz, Germany

**Keywords:** Nanofibre adhesion, Viscous glue, CHCs, Cuticular hydrocarbons, Cribellate, Ecribellate, Prey capture, Evolutionary arms race

## Abstract

Spiders are prominent predators for insects, with which they have a close co-evolutionary history. Manifold capture techniques have evolved, with spider webs being one of most well-known traps in the world. Many webs include specialised threads, bearing either glue or cribellate nanofibres as adhesive to capture prey. Some webs, such as the sheet webs of Tarantulae, have no such intricate threads. The adhesion of gluey threads has been extensively studied already, but often on artificial surfaces. However, recent studies discovered that adhesion of cribellate nanofibres increases massively after contact with insect cuticular hydrocarbons (CHCs). This raises the question whether insect CHCs generally influence prey capture. We compared the adhesion of cribellate, ecribellate gluey and ecribellate non-specialised threads to either uncoated or CHC-coated foil, or native prey body surfaces. We found an influence of CHCs on all silken threads, but with different outcomes. CHC presence, its composition as well as the surface structure can impact the final adhesion force positively or negatively, depending on the thread type. In extreme cases, the adhesion was reduced to nearly zero (e.g. for gluey capture threads in contact with real prey). Thus, prey influence on adhesion is not limited to cribellate capture threads, but is a universal influence on adhesion of spider silken capture threads. Future studies should consider both insect surface chemistry and surface structure when assessing the effectiveness of capture thread types in an ecological and evolutionary context.

## Teaser

**Highlighted Article:** The adhesive properties of different spider capture threads (cribellate, gluey and non-specialised silk) are influenced by the surface chemistry and structure of the prey insect.

## INTRODUCTION

With over 51,000 species, spiders exhibit remarkable diversity, including in their silk characteristics. Spider silk has garnered interest for its mechanical properties and its various applications in biomimetics and medicine (Blamires et al., 2020; Blamires, 2022; Humenik et al., 2018; Borkner et al., 2019; Luken et al., 2021). However, for spiders, it is most important that the silk fulfils its biological function; that is, to successfully adhere to and retain prey. Adhesion is a critical aspect of spiders' prey-capture strategies, and among web-building spiders, various mechanisms have evolved that achieve high adhesion forces with their capture threads. Based on these threads, spiders are divided into cribellate and ecribellate species, with ecribellate spiders having either gluey capture threads or non-specialised threads without any glue, or being active hunters. Cribellate spiders, in contrast, produce composite fibre threads that contain nanofibres with adhesive function (Peters, 1987; Joel et al., 2015; Bott et al., 2017; Opell, 1994; Foelix, 2011).

Researchers have been fascinated by the diversity of ways of creating successful traps in cribellates and ecribellates. The unequal distribution of diversity within all spiders suggests that attributes of their capture threads may have affected diversification of spider taxa (about 9% cribellate, 26% gluey and 65% other ecribellate) (Foelix, 2011; Hormiga and Griswold, 2014). Cribellate spiders spin a composite consisting of up to seven fibre types (Joel et al., 2015; Grannemann et al., 2019; Peters, 1984), each with different mechanical and adhesive properties (Hsiung et al., 2014; Michalik et al., 2019). Thread adhesion is ensured by mechanical interlocking of fine nanofibres with surface structures of prey (e.g. setae), van der Waals forces and, above all, an interaction between the nanofibres and cuticular hydrocarbons (CHCs) that cover the integument of nearly all insects (Bott et al., 2017; Opell, 1994; Hawthorn and Opell, 2003; Joel et al., 2022). In contrast to cribellate threads, gluey capture threads have a less sophisticated structure, but are chemically quite complex: here, the axial fibres are coated with a layer of viscous, aggregated glue that self-assembles into droplets composed of an aqueous layer including inorganic and organic compounds to adjust adhesion properties (Vollrath et al., 1990; Townley et al., 1991; Opell et al., 2017, 2018a,b; Singla et al., 2018). Interspecific differences are mainly found in the spacing and size of these droplets (Opell and Hendricks, 2009). Additionally, viscosity and droplet size adjust to climatic conditions to maximise adhesion (Opell et al., 2013; Amarpuri et al., 2015). Both previously described capture threads vary immensely from those found in other ecribellate weaving spiders. As a prime example, the threads of Tarantulae do not have any known adhesive properties and/or mechanisms. Therefore, they do not have proper ‘capture threads’ that differ from the rest of the web. It was proposed that their webs simply make escape more difficult by being spongy and pliable, which in turn makes it more difficult for prey to walk or launch themselves into flight (Gertsch, 1949; Park and Moon, 2002; Davis and Russell, 1969). The main focus of debate about the superiority of adhesive properties has so far included only cribellate and gluey capture threads. Differences in, for example, extensibility or adhesiveness were invoked to explain the success (measured as diversity) of ecribellate spiders. In most cases, ecribellate gluey capture threads were considered more successful: their silk reduces the reflection of ultraviolet light (and thus decreases their visibility to insects) (Craig et al., 1994) and they achieve higher overall stickiness with better material economy (Chacon and Eberhard, 1980; Opell, 1997). Furthermore, gluey capture threads offer an improvement because of the anchoring of adhesive droplets onto the axial fibres, resulting in a transfer of force called the suspension bridge mechanism (Opell and Hendricks, 2010).

However, to measure adhesion of spider webs, researchers have rarely used the actual prey (i.e. insects) but instead have measured adhesion to smooth artificial surfaces, such as glass or metal, for easier comparisons (for exceptions, see Bott et al., 2017; Eisner et al., 1964; Opell and Schwend, 2007). The adhesion force of cribellate capture threads, though, mainly depends on the interaction between the thread and insect CHCs (Bott et al., 2017). Strong adhesion forces develop because the insect CHCs migrate into the cribellate threads. The migration rate differs between different CHCs, such that the quantitative composition of CHCs that migrate into the threads differs from that on the insect cuticle (Joel et al., 2022). A distinct quantitative composition should yield a viscosity of the CHC mixture that differs from that of the original, potentially influencing adhesion. A detrimental effect of the insect viscous CHC coating, in contrast, was postulated by Opell and Schwend (2007) on gluey capture threads. Insect CHCs are indispensable for insects and fulfil various functions including waterproofing and communication (Blomquist and Bagnères, 2010). The physical properties of the CHC layer, such as viscosity and melting range, are influenced by the qualitative and quantitative CHC composition on the cuticle (Menzel et al., 2019; Gibbs, 2002). While insect *n*-alkanes can aggregate tightly as a result of van der Waals forces and are solid at room temperature, hydrocarbons with one or more methyl branches (methylalkanes) as well as unsaturated hydrocarbons (alkenes or alkadienes) aggregate less tightly and therefore melt at lower temperatures and/or decrease the viscosity of the liquid phase of the CHC layer (Gibbs, 2002; Gibbs and Pomonis, 1995; Sprenger and Menzel, 2020). However, the exact phase behaviour of a CHC layer is still little understood and depends strongly on the inter-molecular interaction between different hydrocarbon molecules (Menzel et al., 2019; Baumgart et al., 2022b). CHC composition varies greatly among species, while within species, the variation is mainly quantitative, i.e. they share different amounts of the same hydrocarbons. Therefore, physical properties of the CHC layer are likely to vary strongly between insect species (Menzel et al., 2019; Baumgart et al., 2022b). Initial studies have indeed indicated an influence of CHC composition and amount on the interaction with cribellate threads (Bott et al., 2017; Joel et al., 2022).

Current hypotheses about the evolution of spider capture threads place the Cribellatae as ancestral to Ecribellatae. Cribellate threads are presumably the first threads that evolved specifically to capture prey, and gluey capture threads have evolved later, being more effective for capturing prey (Dimitrov et al., 2017). Thus, the interaction of spider threads with insect CHCs should be ancestral. This raises the question whether ecribellate gluey threads also interact with insect CHCs, which may support insect capture. If this is true, spider threads may have a long history of exerting selection pressure on insect CHCs and hence influencing their evolution. Therefore, here we performed a comprehensive screening of the interaction of spider capture threads from various taxa with prey insects covered with species-specific CHC profiles. We aimed to study how widespread the interaction of CHCs with spider threads is across thread types, but also across CHC profiles. Thus, we asked how CHC–spider thread interactions vary across insect species and across spider species. The interaction was assessed on both native insects (including surface structures, such as setae) and artificial CHC-coated surfaces. By investigating and comparing the adhesion of spider capture threads and the role of CHCs in this process, we aimed to enhance our understanding of spider–prey interaction. The findings offer additional insights into the co-evolutionary dynamics between spiders and their prey and may help explain the co-existence of different capture threads, each suited to the specific ecological niches realised by their prey.

## MATERIALS AND METHODS

### Ethics

The species used in the experiments are not an endangered or protected species. All applicable international, national and institutional guidelines for the care and use of animals were followed.

### Study animals

For the experiments, three cribellate species [*Amaurobius* sp., *Badumna longinqua* (L. Koch 1867) and *Uloborus plumipes* Lucas 1846], two ecribellate species with gluey capture threads [*Araneus diadematus* Clerck 1757 and *Zygiella x-notata* (Clerck 1757)] and one without specialised capture thread [*Cyriocosmus elegans* (Simon 1889)] were used. *Amaurobius* sp., *A. diadematus* and *Z. x-notata* were caught in the wilds of Aachen (Germany) and *U. plumipes* in garden centres in Aachen. *Cyriocosmus elegans* was bought at a pet shop (Zoohaus W&S, Ludwigshafen, Germany). *Badumna longinqua* was caught in Sydney and Brisbane (Australia) and offspring of these spiders were used in the experiments. Export permission for *B. longinqua* was kindly granted by the Department of the Environment and Energy of the Australian Government (PWS2019-AU-000248). Some experiments with *B. longinqua* were performed either with silk bought at Spider&Silk Supply (Taichung City, Taiwan; webs collected in Sydney, Australia) or with silks produced by *B. longinqua* from New Zealand. Body sizes (prosoma and opisthosoma) between spider species range from about 10 mm (*Amaurobius* sp., *U. plumipes*) to 15 mm (*B. longinqua*, *Z. x-notata*) and up to 20 mm (*A. diadematus*, *C. elegans*).

All species were raised separately under room temperature (ca. 21°C), humidity (ca. 30% relative humidity, RH) and Central European diurnal rhythm. They were fed once a week with either crickets or flies. Water was provided once to twice per month by sprinkling the enclosure. Such wetted webs were not used for further research. Note that humidity has been described to influence the production and functionality of viscous glue droplets, such as those of *A. diadematus* and *Z. x-notata* (Edmonds and Vollrath, 1992; Opell et al., 2017, 2018b).

**Fig. 1.**
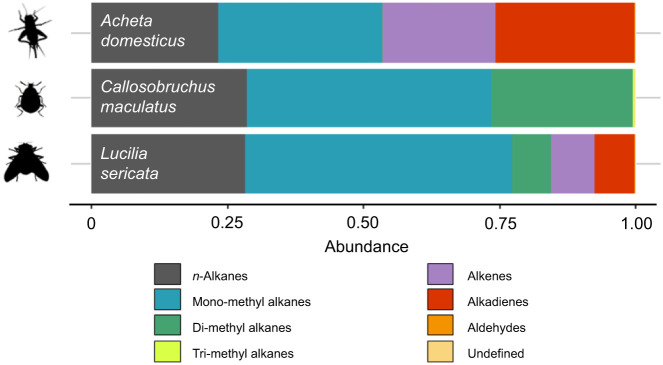
**Overview of the cuticular hydrocarbon (CHC) profiles of the three insect species used in this study.** Data published in Baumgart et al. (2022a).

Insects were selected so that the species were in the potential prey spectrum of the spiders, had as different CHC profiles as possible, and were convenient to obtain and maintain. The cowpea weevil *Callosobruchus maculatus* (Fabricius 1775), the common green bottle fly, *Lucilia sericata* (Meigen 1826), and the house cricket *Acheta domesticus* (Linnaeus 1758), were chosen. The CHC profiles (Fig. 1) have been published in Baumgart et al. (2022a). All three insects have *n*-alkanes and monomethyl alkanes. While the *A. domesticus* profile also contains large proportions of alkenes and alkadienes, *C. maculatus* completely lacks these two CHC classes, but contains dimethyl alkanes, which are absent in *A. domesticus*. *Lucilia sericata* is roughly in between, with smaller proportions of alkenes, alkadienes and dimethyl alkanes. All insects were bought at b.t.b.e. Insektenzucht GmbH (Bad Wörishofen, Germany). All animals were kept at room temperature (ca. 21°C) and room humidity (ca. 30% RH) for at least 1 week before use in experiments.

### Retention of prey

The retention measurements were carried out at room temperature and room humidity (ca. 21°C, ca. 30% RH). For small insects (<0.5 cm), one thread and for large insects (>0.5 cm), two threads (0.5 cm apart) were placed on a bent paper clip. For spiders lacking individual capture threads (such as *C. elegans*), a part of the sheet web (width ca. 0.5 cm) was cut off and placed on a bent paper clip. A living insect was then placed into the threads from above and filmed with a camera (Sony FDR-AX33 Handycam, Sony Group Corp., Tokyo, Japan; or Logitech BRIO 4k Ultra HD Webcam, Logitech international S.A., Apples, Switzerland**)**. If the insect was still unable to flee after 3 min, filming was stopped. Capture threads from *Amaurobius similis* (Blackwall 1861), *C. elegans*, *U. plumipes* and *Z. x-notata* were tested against *A. domesticus* and *C. maculatus* (see Table S1 for detailed replicate number).

The data were analysed for each insect separately using a Cox mixed-effects model with spider species as the explanatory variable and spider individual as a random effect. The effect of the explanatory variable was tested with type-II ANOVA. Pairwise comparisons were done using Tukey *post hoc* comparisons. Analyses were conducted using the R version 4.2.1 on Windows 10 x64 (build 19045) (http://www.R-project.org/).

### Preparation of coated surfaces

For the coating extracts, the insects were covered with *n*-hexane in a 50 ml screw-top bottle (Schott AG, Mainz, Germany) and allowed to stand for 10 min on the rotator. The supernatant was pipetted off and the specimens were again covered with *n*-hexane. After another 10 min, the supernatants were combined. The extract was concentrated to 15 ml. For every 15 ml of extract, we used 187 *A. domesticus*, 300 *C. maculatus* and 249 *L. sericata*. This number is based on the amount of CHCs per insect, determined by quantitative GC-MS analysis. We standardised the coatings on the equivalent of 300 *C. maculatus* extracts, as this amount was found to be a sufficient concentration for proper coating of aluminium foil. For coating, the aluminium foils were fixed with tape at the sides on a metal block with 1 cm wide pits and cleaned with acetone (Rotipuran^®^ ≥99.8 %, Carl Roth GmbH + Co. KG, Karlsruhe, Germany). When cleaning the foil, applying light pressure resulted in small (i.e. no deeper than 1 mm) indentations in the aluminium where the metal block pits had been. Two drops of extract were placed in each of the indentations on the foil. The *n*-hexane was allowed to evaporate, leaving only the extracted CHCs as coating on the aluminium foil. The foil was then cut to strips for the adhesion experiments.

### Adhesion measurements

Adhesion force between capture threads and either the foil or insect surface was determined using a digital microbalance to measure the negative weight. The negative weight is a result of lifting the thread with its sample holder off the microbalance as a result of the stickiness between the thread and tested sample (foil or insect).

**Fig. 2.**
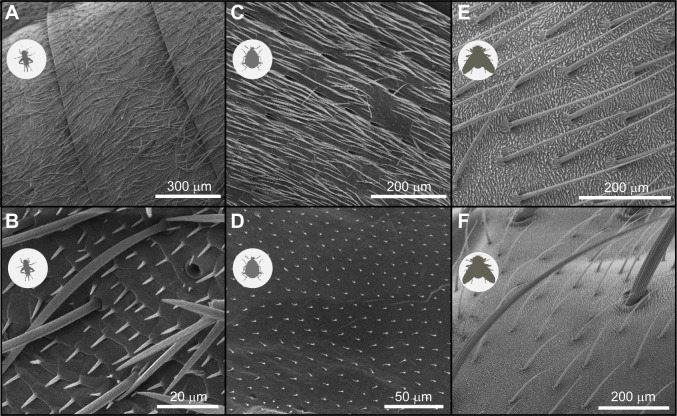
**Different surface structures of the three insect species used in this study.** (A,B) The dorsal side of the abdomen of *Acheta domesticus* (B shows a higher magnification view). (C,D) The elytra (C) and the wing (D) of *Callosobruchus maculatus*. Note that the wing was rarely exposed to the capture threads during adhesion measurements. (E,F) The dorsal side of the abdomen (E) and the thorax (F) of *Lucilia sericata*.

The adhesion measurements were carried out in the controlled environment (28°C and 45% RH) of a climate chamber (HPP IPP^PLUS^, Memmert GmbH+Co. KG, Schwabach, Germany). In experiments testing adhesion to native insects, bent paper clips were used with threads between two parallel wires (distance ca. 8.65 mm) that are part of the clip and wrapped with double-sided adhesive tape. In trials investigating the effect of CHCs alone, a 3D-printed sample carrier with two parallel metal wires (distance ca. 2.6 mm) wrapped with double-sided adhesive tape was used so that the spanned threads connected both parallel arms. In both cases, we confirmed the integrity of the threads prior to experiments under the stereomicroscope (×40). The thread sample carrier was then set onto a microbalance (JB1603/C-FACT; Mettler Toledo, Greifensee, Switzerland), which was placed underneath an arm of a motorised linear table. Both were connected to a computer to measure position, velocity and adhesion force (as a measurement of weight). The sample that was to adhere to the thread was attached to the arm of the linear table. This could be either native insects or CHC-coated aluminium foil wrapped around a metal wire with a round edge (ca. 0.8 mm). Native insects were glued to a toothpick with their dorsal thorax exposed to the threads. In the case of *L. sericata*, the wings were cut off. In the case of *C. maculatus*, both the elytra and thorax could hit the thread. Surface structures differ between the insects (Fig. 2).

The sample was brought in contact with the thread, verified by deflection of the thread and a positive scale response. After 20 s of contact time, the sample was pulled away perpendicular to the capture thread, with a velocity of 1.9 mm s^−1^. The adhesion of the thread could be observed by (a) a deflection of the thread upwards and (b) a negative amplitude of the balance. The adhesion force was then calculated as the measured minimum weight multiplied by gravitational acceleration (Newton's second law of motion; *F*=*m*×*a*). Each sample (insect or foil) and each capture thread was used only once.

The first series of experiments (CHC-coated foil) was analysed by constructing a linear mixed-effects model with insect species and spider species as fixed factors, and spider individual as random factor. Because of an interaction between insect and spider species, we created models separately for each spider species (Fig. S1). Data were tested for normality using the Shapiro–Wilk test (Table S4) and homoscedasticity using Levene's test (Table S4). Although both assumptions were mostly not met, a visual inspection of the histograms and boxplots of the model residuals (Figs S1 and S2) did not indicate a specific bias in any direction. As linear mixed effects models are supposed to be robust to such violations (Zuur et al., 2010; Schielzeth et al., 2020), the results of the models were trusted. Pairwise comparisons were done using a Tukey *post hoc* test. The second series of experiments (native insects) was analysed by constructing a linear mixed-effects model with insect and spider species as fixed factors, and spider individual as random factor. Afterwards, insect-specific models were created. Pairwise comparisons were done using a Tukey *post hoc* test.

## RESULTS

### Cribellate threads retain insects longer than ecribellate threads

**Fig. 3.**
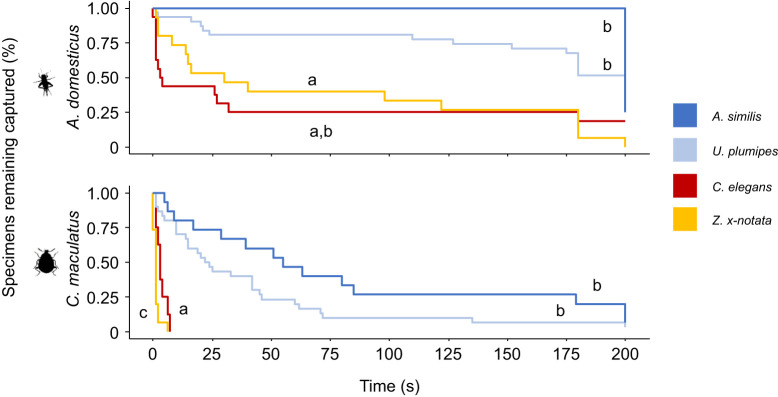
**Prey retention of different spider capture threads.** Data are for capture threads of two cribellate species (*Amaurobius similis* and *Uloborus plumipes*, blue) and one ecribellate species with gluey threads (*Zygiella x-notata*, yellow). Additionally, prey retention was measured for pieces of the sheet web of the tarantula *Cyriocosmus elegans* (red), which lacks specialised capture threads. Different letters indicate statistically significant differences according to a Tukey *post hoc* test. Replicate numbers range from 8 to 31. See Table S1 for statistics and replicate number.

The type of capture thread had a strong effect on the time an insect needed to free itself (‘retention time’). Both gluey and non-gluey ecribellate capture threads performed significantly worse than the cribellate threads in holding insect prey (Fig. 3). This result contrasts with common belief, as gluey capture threads are often assumed to out-perform all other webs. However, prey retention can be influenced by many different factors, including escape behaviour, strength or hairiness of the insects. Hence, we continued our experiments by reducing the interaction to its basis, i.e. a coating of smooth artificial surfaces with the CHCs of different prey.

### CHCs influence adhesion to all spider silks

**Fig. 4.**
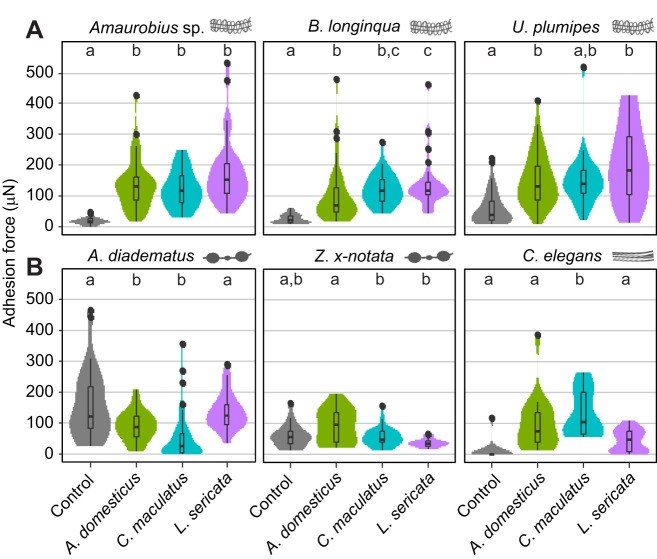
**Adhesion force of capture threads to CHC-coated aluminium foil.** The coating was standardised by CHC amount per individual. Control indicates foil with no coating. Data are for (A) three cribellate species (*Amaurobius* sp., *Badumna longinqua* and *Uloborus plumipes*) and (B) three ecribellate ones, two with gluey capture threads (*Z. x-notata* and *Araneus diadematus*) and one without any specialised capture threads (*C. elegans*). The boxplots illustrate the adhesion forces (µN) of capture threads from different spider species on CHC-coated aluminium foil. Each boxplot displays the median (horizontal line within the box), the lower (Q1) and upper (Q3) quartiles, and the whiskers, which extend to the last data point within 1.5 times the interquartile range (IQR). Data points outside this range are shown as individual outliers. Additionally, the violin plots surrounding the boxplots depict the distribution density of the measurements. Different letters within each plot indicate statistically significant differences according to a Tukey *post hoc* test. Replicate numbers range from 15 (only samples from *C. elegans*) to 34–35 (all others). See Table S2 for replicate number and Table S3 for statistical results.

For all spider species, excluding those with gluey capture threads, adhesion force could significantly increase for CHC-coated foil than for non-coated controls (Table S3; Fig. 4). An effect of chosen insect species, however, was only detected in one case, for threads of *B. longinqua* in contact with foil coated with CHCs from *A. domesticus* or *L. sericata*. This is contrasted by ecribellate silk: gluey capture threads performed equally well (*Z. x-notata*) or even worse (*A. diadematus*) on CHC-coated surfaces, with the CHCs of one insect adhering significantly better than those of the other two (for *A. diadematus* to *L. sericata*; and for *Z. x-notata* to *A. domesticus*). Surprisingly, the cut-out sheets of the web of the tarantula *C. elegans* did not perform worse than cribellate capture threads, and they sometimes performed even better than gluey capture threads (e.g. for *C. maculatus* CHC-coated foil χ^2^=45.097, *P*<0.001). The CHC coatings of *A. domesticus* and *L. sericata* did not affect adhesion in comparison to the CHC-free control foil. However, the CHCs of *C. maculatus* did result in a significantly higher adhesion of the foil to the webs of *C. elegans*.

Our data did thus confirm the superior adhesion force of gluey capture threads to non-coated surfaces, comparing the adhesion force of the different capture threads to these samples. However, on CHC-coated surfaces, the adhesion force of gluey capture threads was often worse than that for the non-coated surfaces. Their adhesion force was outperformed by cribellate capture threads as well as webs of a tarantula, depending on the insect species chosen for coating (Table S3). It is astonishing to detect a CHC-driven adhesion increase in the webs of *C. elegans*, a spider building webs mimicking the hypothetical first webs produced by spiders (Coddington et al., 2019; Kulkarni et al., 2023). This increase, though, was not always significant.

### Visual signs of interaction

**Fig. 5.**
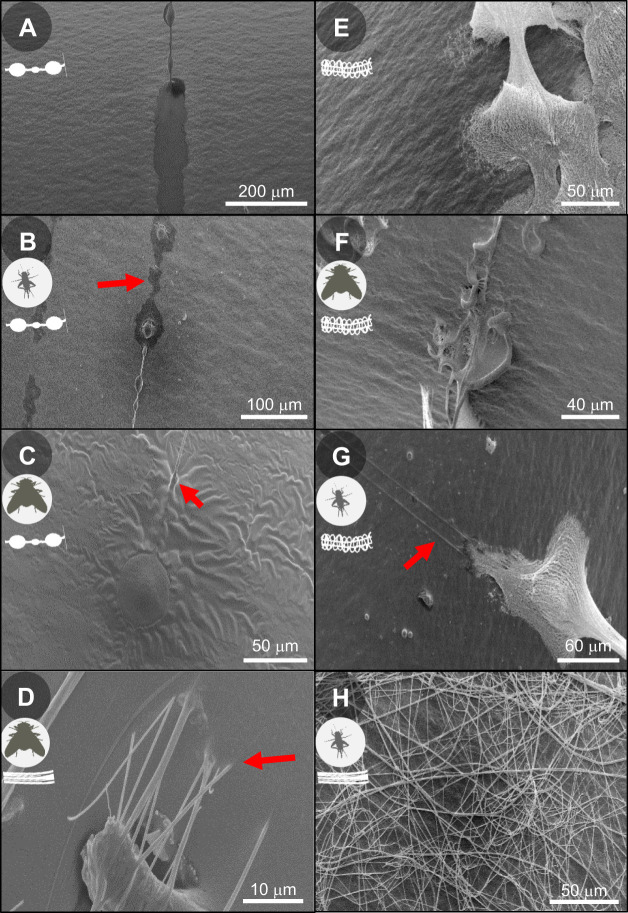
**Interaction of spider silks with the CHCs of insect prey.** (A) A thread of *Z. x-notata* in contact with uncoated foil. (B) A *Z. x-notata* thread in contact with a strip of foil previously coated with *A. domesticus* CHCs. The red arrow marks the region where the spreading of the droplet of glue was reduced. (C) An *A. diadematus* thread in contact with a foil strip coated with *L. sericata* CHCs. The red arrow points to a likely upwards migration of CHCs on the fibres of the gluey capture thread. (D) A *C. elegans* web in contact with a foil strip previously coated with *L. sericata* CHCs. Some parts of single threads are embedded in the layer of CHCs (red arrow). (E) A *U. plumipes* thread in contact with uncoated foil. (F) A *B. longinqua* thread in contact with a foil strip coated with *L. sericata* CHCs. (G) A *U. plumipes* thread in contact with a foil strip coated with *A. domesticus* CHCs. The red arrow highlights the two axial fibres of the cribellate thread which remain visible. (H) A *C. elegans* web in contact with a foil strip coated with *A. domesticus* CHCs. Threads rest on top of the CHCs and show no sign of interaction.

Upon visual investigation, the most pronounced reaction occurred when cribellate threads were brought into contact with CHC-coated surfaces (Fig. 5D,E,F). Interestingly, the behaviour of other silk types differed; whereas droplets spread widely on uncoated surfaces (Fig. 5A), they became encapsulated on coated ones (Fig. 5B). In certain cases, it even appeared as though CHCs migrated upwards and into the gluey capture threads (Fig. 5C). In some cases, fibres of *C. elegans* also embedded into the CHC, but not always or as prominently as for cribellate threads (Fig. 5G,H).

### Adhesion to natural prey

**Fig. 6.**
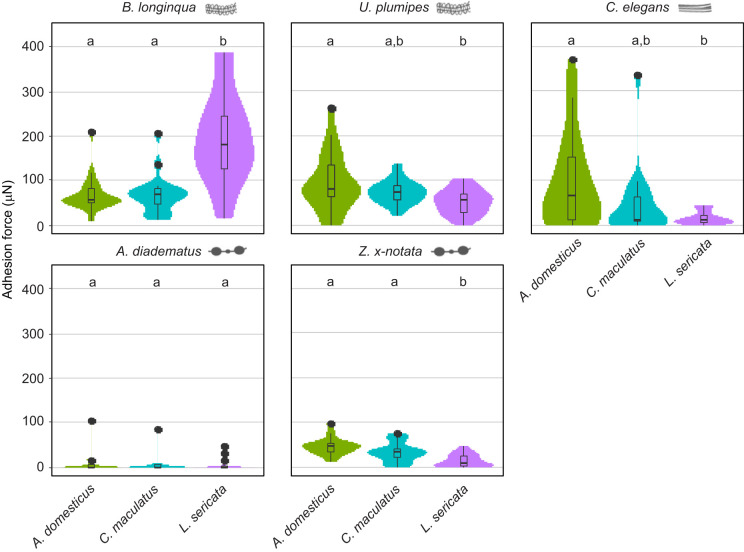
**Adhesion of spider silks to insect body surfaces.** Data are for two cribellate species (*B. longinqua* and *U. plumipes*) and three ecribellate ones (*A. diadematus*, *Z. x-notata* and *C*. *elegans*). The boxplots illustrate the adhesion forces (µN) of capture threads from different spider species on insect body surface. Each boxplot displays the median (horizontal line within the box), the lower (Q1) and upper (Q3) quartiles, and the whiskers, which extend to the last data point within 1.5 times the interquartile range (IQR). Data points outside this range are shown as individual outliers. Additionally, the violin plots surrounding the boxplots depict the distribution density of the measurements. Different letters within each plot indicate statistically significant differences according to a Tukey *post hoc* test. Number of replicates ranges from 15 (*C. elegans* and *Z. x-notata*) to 18–19 (all others). See Tables S2 and S3 for details.

A coating with CHCs influenced the interaction of spider capture threads with prey. Hairs and other surface features of insects are likely to influence the adhesion force between prey and spider capture threads, too. Hence, we validated our results for three insect species, attaching the thread to the dorsal thorax part of the bodies. Here, the previously described decreased adhesion force of gluey capture threads was even more pronounced, with the capture threads of *A. diadematus* almost not adhering to the prey at all (Fig. 6). In general, the threads of cribellate species adhered better than gluey capture threads, and demonstrated varying levels of adhesion with native prey. This behaviour was not uniform between the two cribellate species, with *B. longinqua* adhering best to *L. sericata*, while *U. plumipes* adhered best, though not significantly, to *A. domesticus*. Again surprisingly, the tarantula *C. elegans* showed comparable adhesion force to that of cribellate capture threads, reflecting the data generated with the coated foil.

## DISCUSSION

**Fig. 7:**
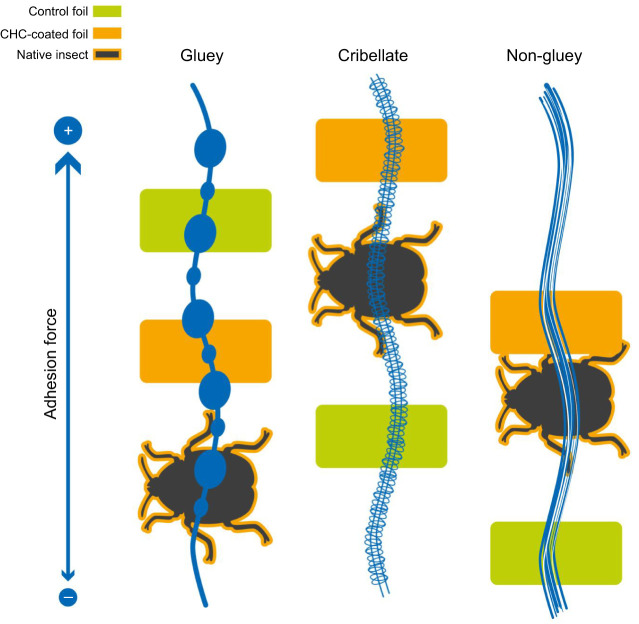
**. Differences in adhesion of spider capture threads to artificial surfaces or real prey.** Data are for gluey ecribellate, cribellate and non-gluey ecribellate threads adhering to control foil, CHC-coated foil or prey and were combined for spider species and insect prey; thus, this graphical summary simplifies the data presented in Figs 4 and 6, and the corresponding statistical data presented in Tables S2 and S3.

The comparison between cribellate and ecribellate spiders has attracted many researchers, as there are far more species with gluey than with cribellate capture threads. So far, most studies described the gluey capture thread to perform better, indicating that cribellate spiders could prevail only under specific climatic conditions. With few exceptions, though, all adhesion tests were performed on non-biological CHC-free surfaces and adhesion under different climatic conditions might be influenced differently on more natural surfaces. While analysing the adhesion of capture threads of different species, cribellate and ecribellate, to real prey or prey-mimicking surfaces, we could not reproduce the superiority of gluey capture threads but in fact observed higher or comparable adhesive forces for cribellate threads. Surprisingly, even the webs of the tarantula species *C. elegans* showed adhesion forces comparable to those of the other species, performing better when in contact with real prey or CHC-coated surfaces. This contrasts with gluey capture threads and reflects the same trend as observed in cribellate threads (Fig. 7).

When comparing the adhesion strength of capture threads of cribellate and ecribellate spiders on CHC-coated surfaces, it was remarkable that (a) CHC coating could decrease adhesion in gluey threads, but increased adhesion in cribellate and non-gluey ecribellate threads, and (b) mainly gluey capture threads showed CHC profile-specific differences (i.e. CHCs of different insects). For cribellate capture threads as well as for the non-gluey web sheet of *C. elegans*, a CHC coating in general had a positive effect on adhesion force. For gluey capture threads, it did not matter whether the smooth surface was coated or not, although there was a tendency for adhesion to decrease on coated surfaces, with differences depending on the type of coating. This effect, though, was strengthened as soon as the thread had contact with natural prey surfaces. Gluey capture threads almost did not adhere at all to native insect surfaces. This observation is intriguing, as it suggests a direct influence of CHCs on the adhesive force for all spider capture threads, which is even more pronounced on natural insect surfaces. As our results on uncoated aluminium foil mimic the results presented in these previous studies (Hawthorn and Opell, 2003; Amarpuri et al., 2015; Eberhard, 1980; Opell, 1989, 1993, 1995; Hawthorn and Opell, 2002; Agnarsson and Blackledge, 2009; Piorkowski et al., 2021), our results stress the importance of performing future experiments of spider thread adhesion using surfaces that closely mimic natural conditions.

### Adhesion of cribellate capture threads

The cribellate adhesion mechanism relies heavily on CHCs and previous studies predicted adhesion to be influenced by CHC composition (Bott et al., 2017; Joel et al., 2022). It is surprising that these capture threads adhered equally well despite varying CHC profiles on artificial surfaces in this study. This result, though, was contrasted by the results with natural prey, where adhesion force differed significantly across the different insect species. It has already been suggested that the hairiness of prey influences adhesion of cribellate threads (Opell, 1994). This could at least partly explain the differences between the coated foil and natural prey. However, threads of the two species *U. plumipes* and *B. longinqua* reacted differently to the native insects of the same prey species, suggesting surface structures can only partly explain the differences. As these differences are not reflected in the coated foil experiments, they are difficult to explain. Possibly, they are caused by structural and chemical differences of the threads in the two species (Joel, 2016; Weissbach et al., 2021; Joel et al., 2023).

### Adhesion of gluey capture threads

In contrast to the expected high performance of gluey capture threads, these threads performed worst on natural prey and showed slightly inferior performance when a smooth surface was coated with CHCs in comparison to control foil. The lower efficiency on coated surfaces, though, might be due to their exceptional adhesion to the control sample without coating. A reduced adhesion to hairy prey could be explained by the suspension bridge mechanism of gluey capture threads, which is probably minimised by a reduced contact area caused by insect setae (Opell and Hendricks, 2009). In another study, Opell and Schwend (2007) tested the influence of surface features on adhesion and describe that setal length and area in combination with the glue droplet volume influence adhesion force to insects. They argue that insect setae can increase the contact area and thus enhance adhesion. Our observed reduced adhesion to insects themselves is, however, reflected in our measured retention time: cribellate capture threads retained insects the longest, while prey escaped from gluey capture threads often within the first few seconds. This is consistent with other studies describing a retention time of less than a second for 25% of all tested insects (Blackledge and Zevenbergen, 2006). In the context of retention time, insects do not need to be captured indefinitely, but just long enough for the spider to notice and attack the insect. For many spiders, this is a time span of 5 to 10 s (Eberhard, 1989; Lubin, 1973). Hence, the extremely long retention of prey in cribellate capture threads described here does not necessarily indicate a prevailing adhesion mechanism. Many other factors, such as web orientation, mesh size, insect impact or restricted movement behaviour of the prey can contribute to successful capture (Craig et al., 1994; Opell and Schwend, 2007; Blackledge and Zevenbergen, 2006; Eberhard, 1989; Zschokke and Nakata, 2015) and might be superior in many Araneidae to that in cribellate spiders. Additionally, it is well described that adhesion of gluey capture threads is influenced by humidity (Opell and Schwend, 2007; Edmonds and Vollrath, 1992; Opell et al., 2021; Amarpuri et al., 2015). The interplay between ambient humidity, droplet spacing, CHCs and insect surface features has not been addressed in our study. The 30% RH used in this study might have been unfavourable for gluey capture threads, as these tend to adhere better at higher humidities (Edmonds and Vollrath, 1992; Amarpuri et al., 2015; Opell et al., 2021). As another study describes gluey capture threads as adhering more uniformly on native insect surfaces compared with cribellate threads of Uloborids (Opell and Schwend, 2007), the interplay between capture threads and prey needs further evaluation to be fully understood.

### Co-evolution of spider webs and insect prey

Detecting a general influence of CHCs, but also an effect of CHC composition on the adhesion of both gluey and non-gluey ecribellate threads raises the question of the evolutionary dynamics of this interplay. In the CHC interaction screening, ecribellate threads did not show the same reaction as cribellate capture threads. Still, for the non-gluey silk sheet of the tarantula, it appeared that some individual fibres were embedded in the CHCs, while most of them were just sitting on top. The observation for gluey capture threads was much more difficult to interpret: it seemed that the CHCs encased the spreading area of the glue. In a few cases, it appeared that the CHCs not only encircled the axial thread but even migrated upwards into the thread, which might be similar to CHC migration into cribellate threads (Bott et al., 2017; Joel et al., 2022) although the diameters of fibres as well as their chemistry differ immensely. The detection of this varying level of interaction between all tested silks and the CHCs of insects still indicates that the evolutionary arms race between spider predator and insect prey very probably influenced spider silk evolution even before the evolution of cribellate capture threads. Thus, the propensity to interact with insect CHCs might be an ancestral trait of spider silk. The CHC profiles of the three insect species differ strongly. *Acheta domesticus* bears the highest proportion of alkenes and alkadienes, which have the lowest melting points, and thus the lowest viscosity of all CHC classes common in insects (Gibbs, 2002). Previous studies with *U. plumipes* threads suggest that CHC viscosity influences the propensity of CHCs to migrate into spider threads, with low-viscosity CHCs migrating the fastest, and highly viscous ones migrating only little (Joel et al., 2022). Hence, the proportion of low-viscosity compounds might influence adhesion forces. However, the adhesion forces we observed here were very specific to spider thread type. Foil coated with *A. domesticus* CHCs did not always induce the highest adhesion forces. Furthermore, *L. sericata* has a CHC class composition that is intermediate between those of the other two species, but instead of inducing intermediate adhesion, foil coated with *L. sericata* CHCs induced the highest adhesion to *B. longinqua* threads and the lowest adhesion to *Z x-notata* threads, compared with the other two insects (Fig. 6). Hence, the CHC–thread interaction may not only depend on CHC composition but also on thread type. The effect of CHC composition on adhesion might differ between thread types, such that each thread type would exert different selection pressures on the insect CHC composition. Finally, the phase behaviour of the CHC layer, such as the solid–liquid distribution and phase heterogeneity, may not only depend on CHC class composition but also on other factors such as chain length distribution, methyl branch positions and number of compounds (Menzel et al., 2019; Baumgart et al., 2022b).

It is astonishing that gluey capture threads adhere much less to insects, not only compared with cribellate threads but also with non-specialised threads of *C. elegans*. This questions the frequently proposed superiority of gluey capture threads over all other capture threads. Cribellate capture thread production is a labour- and time-intensive process (Zschokke and Vollrath, 1995), and so is the production of sheet webs like that of Tarantulae. The strong superiority of prey retention demonstrated by cribellate threads may still point to a valuable investment. However, if there are sufficient prey, the costs saved by using a less energy-intensive thread might be worthwhile and may have favoured the enormous diversification in Araneoidea. Given the prey scarcity due to continuous declines in both abundance and biomass (Wagner et al., 2021), a resulting reduction in the population of spiders equipped with gluey capture threads could ensue. However, as we could detect an influence of CHC composition on the adhesion of ecribellate but not cribellate threads, more ecribellate species and more different potential prey species should be studied to disentangle the co-evolutionary process shaping spider silk and insect prey coating.

### Conclusion

Analyses of adhesion forces of spider capture threads to native insect or smooth CHC-coated surfaces revealed that cribellate and ecribellate capture threads adhered equally well, with gluey capture threads underperforming on prey. Aside from adding this surprising result to the debate on the superiority of gluey capture threads over any other thread type, observing a CHC-dependent adhesion for all spider capture threads strongly indicates a co-evolutionary process between the CHCs covering insects and spiders as predators. The interaction might be a universal feature of spider silk, taken to an extreme by cribellate silk and hampered by the glue droplets of ecribellate spiders. As our observations are the first of their kind, they stress the importance of considering the influence of CHCs on spider silk in future studies. At the same time, this strongly suggests that spider capture threads have exerted selection on insect CHC composition for a long time.

## Supplementary Material

10.1242/jexbio.249347_sup1 Supplementary information
