# Supplementary material for: The sticky truth: how spider predation success depends on their prey's body surface
Source: J Exp Biol. 2025 May 8;228(9):jeb249347. doi: 10.1242/jeb.249347 (PMC12079663; doi:10.1242/jeb.249347)
Supplement: Supplementary information [file jexbio-228-249347-s1.pdf]

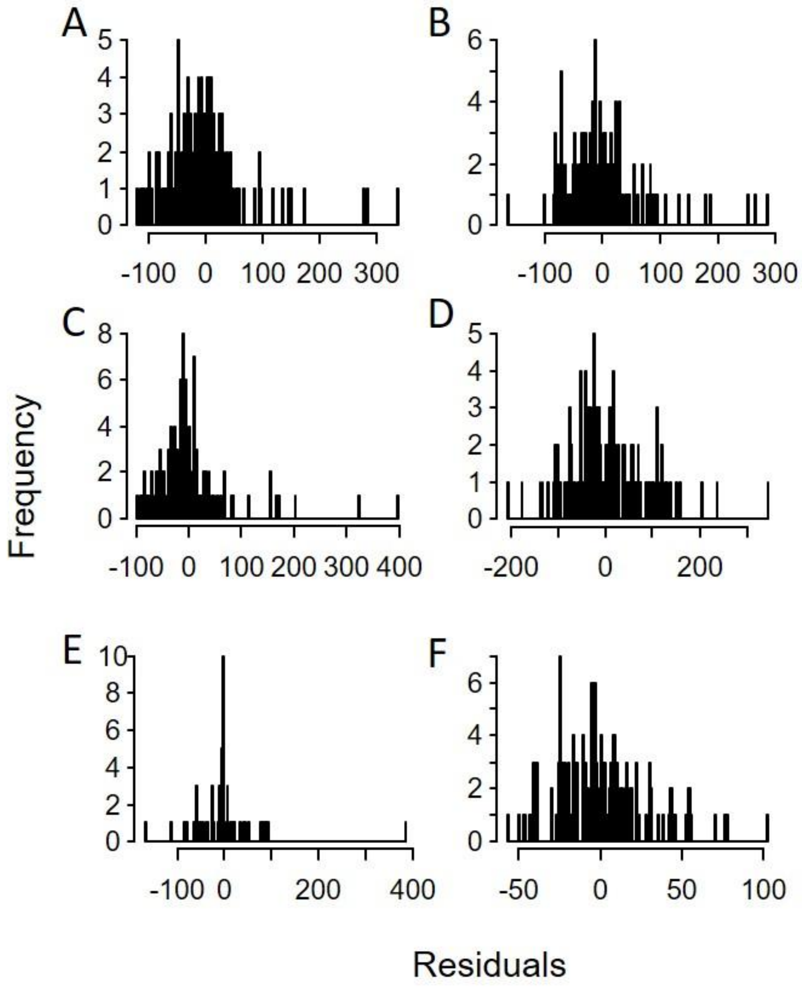

Fig. S1. Histograms of distribution of residuals of linear mixed-effects models investigating adhesion force of spider silk to different surface coatings. A) *Amaurobius* sp. B) *A.diadematus* C) *B. longinqua* D) *C. elegans* E) *U. plumipes* and F) *Z. x-notata*. See also [1, 2] about the robustness of linear mixed-effects models.

Journal of Experimental Biology • Supplementary information

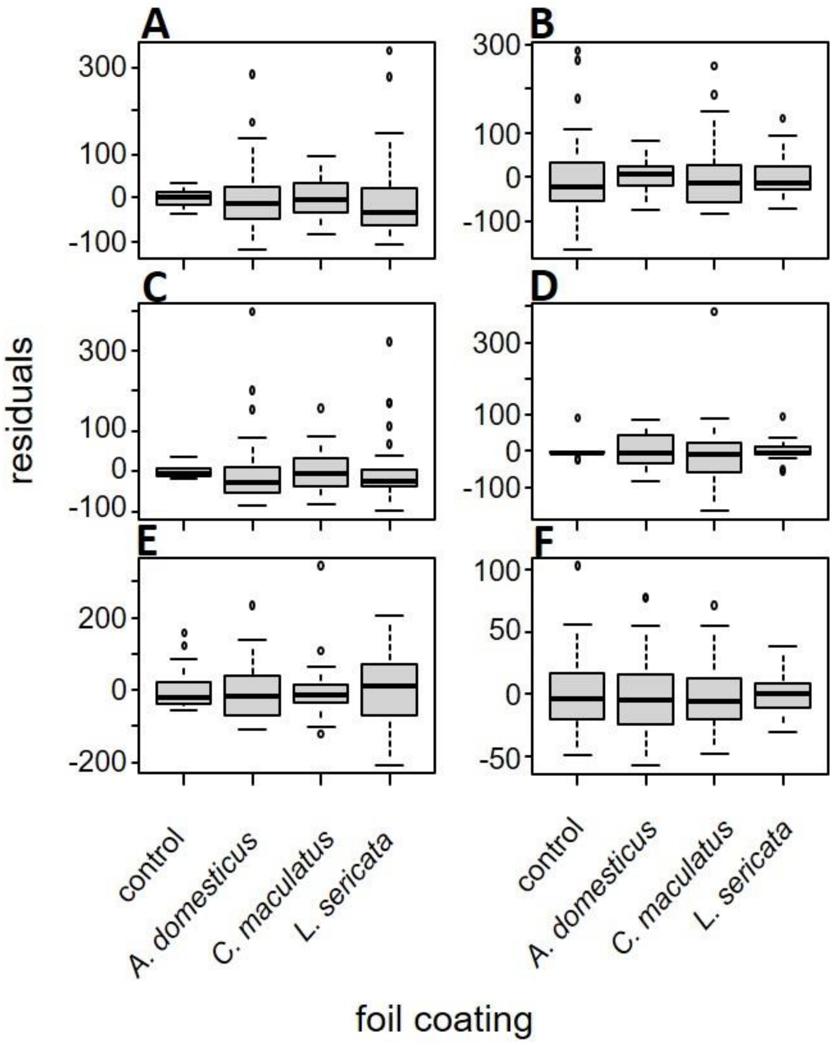

Fig. S2. Boxplot of residuals of linear mixed-effects models investigating adhesion force of spider silk to different surface coatings. Threads of (A) *Amaurobius* sp., (B) *A. diadematus*, (C) *B. longinqua*, (D) *C. elegans*, (E) *U. plumipes* and (F) *Z. x-notata* were brought into contact to foils coated with CHCs of *A. domesticus*, *C. maculatus*, *L. sericata* or uncoated foils (control).

Journal of Experimental Biology • Supplementary information

Table S1. Replicate number and results of the statistical analysis of the retention experiment. The data was analysed for each insect separately using a Cox mixed-effects model with spider species as the explanatory variable and spider individual as a random effect. The effect of the explanatory variable was tested with type-II ANOVA. Pairwise comparisons were done using a Tukey post hoc comparisons.

Replicate number				
	A. similis.	*C. elegans*	*U. plumipes*	*Z. x-notata*
A. domesticus	8	16	31	15
C. maculatus	15	16	30	15

Statistical analysis				
Dataset	fixed factor	df	χ^2	p
A. domesticus	spider	3	15.933	**0.001**
C. maculatus	spider	3	52.841	< **0.001**

Table S2: Replicate number for the adhesion experiments with (non-)coated aluminium foils or native insects.

Foils	Control foil	*A. domesticus*	*C. maculatus*	*L. sericata*
Amaurobius spec.	34	35	35	35
A. diadematus	35	35	35	35
B. longinqua	35	34	34	35
C. elegans	15	15	15	30
U. plumipes	35	35	35	35
Z. x-notata	35	35	35	35
Native prey insects				
A. diadematus		18	18	18
B. longinqua		18	19	18
C. elegans		15	15	16
U. plumipes		18	18	18

Table S3. Results from linear mixed-effects models on adhesion experiments with (non-)coated aluminium foils or native prey insects.

Foils: The first part of the table gives results for a model including all insect and all spider species. "Insect species" includes the control treatment (i.e. no coating) as a fourth factor level. The second part gives results for separate models for each insect species. The third part refers to separate models for each spider species.

Native prey insects: The first part of the table gives results for a model including all insect and all spider species. The second part gives results for separate models for each insect species. *: did not meet assumption of normality.

Foils

Dataset	fixed effect	df	χ^2	p
All data	Insect species	3	87.039	< **0.001**
	Spider species	5	66.500	< **0.001**
	Insect x spider	15	186.403	< **0.001**
Amaurobius spec.	Insect species	3	81.170	< **0.001**
A. diadematus	Insect species	3	42.322	< **0.001**
B. longinqua	Insect species	3	58.078	< **0.001**
C. elegans	Insect species	3	36.778	< **0.001**
U. plumipes	Insect species	3	16.810	< **0.001**
Z. x-notata	Insect species	3	30.799	< **0.001**
Control	Spider species	5	101.780	< **0.001**
A. domesticus	Spider species	5	18.720	0.002
C. maculatus	Spider species	5	45.097	< **0.001**
L. sericata	Spider species	5	35.637	< **0.001**

Native prey insects

Dataset	fixed effect	df	χ^2	p
All data	Spider species	5	6.162	< **0.001**
	Insect species	2	0.032	0.968
	Spider x insect	8	10.281	< **0.001**
*A. diadematus**	Insect species	2	0.239	0.788
B. longinqua	Insect species	2	13.340	< **0.001**
C. elegans	Insect species	2	3.772	0.031
U. plumipes	Insect species	2	4.420	0.017
Z. x-notata	Insect species	2	10.724	< **0.001**

Journal of Experimental Biology • Supplementary information

Table S4. Results of the Shapiro-Wilk normality and Lavene's test.

Shapiro-Wilk	W		p
Amaurobius sp.	0.864		< 0.001
A. diadematus	0.880		< 0.001
B. longinqua	0.756		< 0.001
C. elegans	0.758		< 0.001
U. plumipes	0.953		< 0.001
Z. x-notata	0.960		< 0.001
Lavene's	df	F	p
Amaurobius sp.	3	6.3	< 0.001
A. diadematus	3	4.3	0.006
B. longinqua	3	3.6	0.014
C. elegans	3	4.6	0.006
U. plumipes	3	3.5	0.02

References

1. Zuur, A.F., Ieno, E.N., and Elphick, C.S. (2010). A protocol for data exploration to avoid common statistical problems. Methods in Ecology and Evolution *1*, 3-14.

2. Schielzeth, H., Dingemanse, N.J., Nakagawa, S., Westneat, D.F., Allegue, H., Teplitsky, C., Réale, D., Dochtermann, N.A., Garamszegi, L.Z., and Araya-Ajoy, Y.G. (2020). Robustness of linear mixed-effects models to violations of distributional assumptions. Methods in Ecology and Evolution *11*, 1141-1152.

Journal of Experimental Biology • Supplementary information
